# New Frontiers for Old Medications: Repurposing Approved Drugs Against Gram-Negative Bacterial Infections

**DOI:** 10.3390/microorganisms13092115

**Published:** 2025-09-10

**Authors:** Ronit Aloni-Grinstein, Emanuelle Mamroud, Yoav Gal

**Affiliations:** Department of Biochemistry and Molecular Genetics, Israel Institute for Biological Research, Ness Ziona 7410001, Israel; emmym@iibr.gov.il

**Keywords:** Gram-negative bacteria, repurposed drugs, antibiotic resistance, host-directed therapies, bioterror agents

## Abstract

The global escalation of antimicrobial resistance (AMR) among Gram-negative bacteria poses a severe threat to public health. Traditional antibiotic development struggles to keep pace with emerging resistant strains, necessitating innovative strategies to enhance therapeutic options. This review explores the potential of drug repurposing as a strategic approach to combat Gram-negative bacterial infections, focusing on clinically approved drugs with antibacterial properties or the capacity to enhance antibiotic efficacy through direct or host-directed mechanisms. Within the review, a special section is dedicated to the potential usage of repurposed drugs against bacteria that can be used as biological warfare agents, exposure to which may lead to mass casualties, in particular if these pathogens are resistant to antibiotics. Repurposed drugs exhibit diverse antibacterial mechanisms, including membrane disruption, efflux pump inhibition, iron metabolism interference, quorum sensing suppression, and biofilm inhibition. Additionally, many agents demonstrated host-directed therapeutic effects by modulating inflammatory responses, enhancing autophagy, or boosting innate immune functions. Drug repurposing offers a promising avenue to mitigate the AMR crisis by providing rapid, cost-effective therapeutic solutions. Combining repurposed drugs with existing antibiotics or employing them as host-directed therapies holds significant potential for treating infections caused by multidrug-resistant Gram-negative pathogens. Continued research and clinical validation are essential to translate these findings into effective treatment regimens.

## 1. Introduction

### 1.1. Clinical Burden of AMR

Antimicrobial resistance (AMR) among Gram-negative bacterial pathogens represents one of the most pressing health threats worldwide. Organisms such as *Escherichia coli*, *Klebsiella pneumoniae*, *Pseudomonas aeruginosa*, and *Acinetobacter baumannii* are frequent causes of severe infections in both healthcare and community settings. While plant pathogens such as *Xanthomonas campestris* and *Pseudomonas syringae* exhibit resistance to streptomycin, highlighting how antibiotic use in farming drives resistance selection [1], environmental bacteria, including *Burkholderia cepacia complex* and *Pseudomonas putida*, naturally harbor a vast reservoir of resistance genes (“the resistome”) that can potentially transfer to human pathogens under antibiotic pressure [2,3]. Waterborne organisms like *Legionella pneumophila* and *Vibrio vulnificus* are also showing emerging patterns of resistance [4,5]. Furthermore, bacterial species of bioterrorism concern, such as *Bacillus anthracis*, *Yersinia pestis*, *Francisella tularensis*, *Coxiella burnetii*, *Burkholderia mallei*, and *Burkholderia pseudomallei*, although traditionally treatable, pose heightened risks if engineered to carry antibiotic resistance traits [6]. These interconnected reservoirs across clinical, agricultural, and environmental domains emphasize the urgent need for an arsenal of medications. The rise in multidrug-resistant (MDR) and extensively drug-resistant (XDR) strains has increased mortality, prolonged hospitalizations, and escalated healthcare costs.

### 1.2. Rationale for Drug Repurposing

The traditional antibiotic pipeline is slow, costly, and unable to keep pace with the speed at which resistance emerges. Repurposing of existing, clinically approved drugs offers a cost-effective and rapid alternative strategy. These agents already have well-characterized pharmacokinetics and safety profiles, thereby accelerating translation into clinical use. Diverse drug classes, including non-steroidal anti-inflammatory drugs (NSAIDs), anticancer agents, statins, selective serotonin reuptake inhibitors (SSRIs), cardiovascular medications, antifungals, and antiparasitics, have demonstrated direct antibacterial activity or the ability to potentiate existing antibiotics. Their reported effects include disruption of bacterial membranes, inhibition of efflux mechanisms, interference with iron metabolism, quorum-sensing suppression, and biofilm inhibition. Moreover, many of these agents modify host immune responses, acting as host-directed therapies (HDTs) by reducing inflammation, modulating inflammasome activity, or enhancing bacterial clearance.

### 1.3. Novelty and Scope of This Review

This review highlights recent advances in repurposing clinically approved drugs against Gram-negative bacterial infections. We begin by sharing features and outlining the common antibacterial properties observed within each major pharmacological group, and then emphasize drug-specific findings to avoid redundancy. Particular attention is devoted to the dual role of certain drugs: not only do they exhibit antibacterial properties, but they also function as host-directed or immunomodulatory therapies. A dedicated section addresses the potential use of repurposed drugs against pathogens of concern for biological warfare (all of which, except *Bacillus anthracis*, are Gram-negative bacteria), where alternative treatments are urgently needed if conventional antibiotics fail. Collectively, these insights underscore the potential of drug repurposing as a timely and effective strategy to combat the AMR crisis.

## 2. Host-Directed Therapies (HDT) in Infectious Diseases

Immunotherapies targeting infectious diseases, known as host-directed interventions, aim to modify intracellular processes or immune system responses (both innate and adaptive) to microbial pathogens. These interventions can enhance immune efficacy against pathogens, impair virulence factors, and/or prevent immunopathology (e.g., inhibiting excessive inflammatory responses). Host-Directed Therapy (HDT) presents an alternative approach to direct antibacterial treatment, with a key advantage being the reduced likelihood of resistance development, particularly for therapies targeting multiple cellular mechanisms essential for microbial pathogenesis. HDT can encompass safe treatments, often based on natural compounds, vitamins, and dietary supplements. Another advantage of HDT is its potential for concurrent use with other antibacterial agents (including antibiotics, repurposed drugs, antibodies, etc.) [7,8]. Skyberg [9] extensively detailed immune potentiation against threat-listed bacteria a decade ago, addressing each bacterium across various exposure routes. While many treatments described in earlier reviews (e.g., TLR agonists, CpG, interferons) were not approved, current clinical trial pipelines include numerous studies examining therapies based on these mechanisms [10]. HDT based on modulating inflammatory responses and immune potentiation has been recommended as an antibacterial strategy, including against threat-listed and other Gram-negative bacteria. Tools for identifying novel host proteins have been proposed to design small host-directed molecules for the treatment of bacterial infections [11]. Extensive literature on HDT potential focuses on *M. tuberculosis* [7,12], which may be relevant for treating other intracellular bacteria, including those on threat lists. This applies to other intracellular bacteria for which effective, low-toxicity drug treatments have been proposed [13]. The following section will provide examples of drugs operating via HDT mechanisms, highlighting their potential in combating bacterial infections, particularly those caused by resistant strains.

### 2.1. Phosphoinositide 3-Kinase (PI3K) Inhibitors

Pathogenic bacteria utilize phosphoinositide 3-kinase (PI3K) to enhance their uptake into host cells, inhibit phagosome maturation (increasing their survival within macrophages), and prevent lysosomal fusion. This not only protects them from host immune killing but also impairs the efficacy of antibiotic treatment. Consequently, combining PI3K inhibitors with antibiotic therapy may improve treatment outcomes for intracellular bacteria and facilitate more effective bacterial clearance [14].

### 2.2. Thalidomide

Despite thalidomide’s limited efficacy against Gram-negative bacteria (MIC = 712.5 µg/mL for *E. coli*), its immunomodulatory activity as a TNFα inhibitor has demonstrated benefits in rat sepsis models (MDR *P. aeruginosa* or *E. coli*) [15,16].

### 2.3. Drugs Affecting NETosis and Iron Metabolism

Drugs that enhance immune system activity against bacteria or prevent bacterial utilization of critical host-supplied substances, such as iron, may serve as an important therapeutic strategy. These adjuvant therapies could be valuable in combating resistant bacteria, as extensively reviewed in a recent publication [10].

In conclusion, HDT represents a promising strategy for addressing resistant bacteria, particularly when combined with antibacterial treatments. Therefore, we believe it is essential to closely monitor developments in this field.

## 3. Approved Drugs with Antibacterial Activity or Enhancing/Restoring Antibiotic Sensitivity

**Figure 1:**
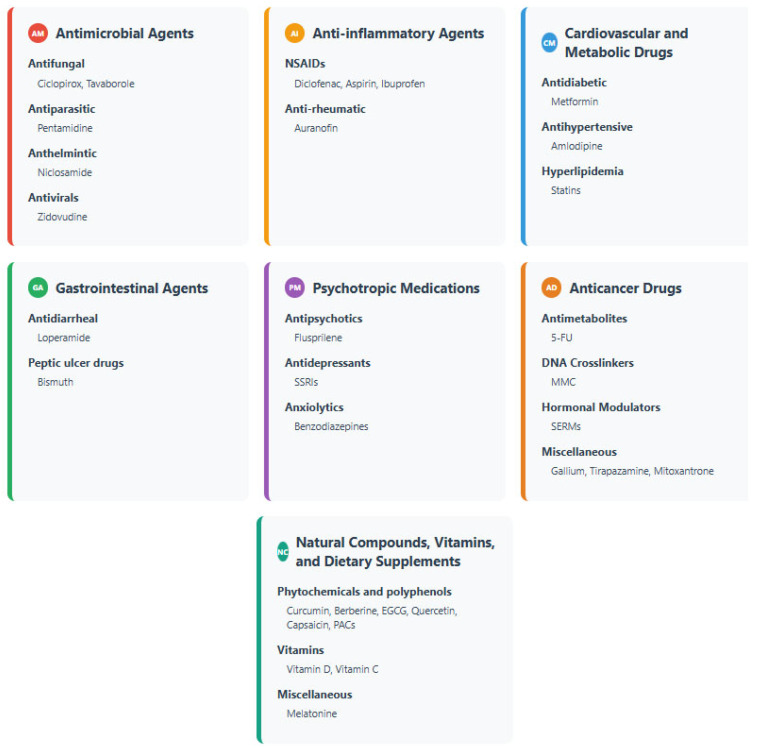
Pharmacological categorization of repurposed drugs exhibiting antibacterial activity against Gram-negative bacteria. EGCG = epigallocatechin gallate; 5-FU = 5-fluorouracil; MMC = mitomycin C; NSAIDs = non-steroidal anti-inflammatory drugs; PACs = proanthocyanidins; SERMs = selective estrogen receptor modulators; SSRIs = selective serotonin reuptake inhibitors.

This section elaborates on repurposed drugs with direct antibacterial activity, or adjuvant effects that enhance existing antibiotic efficacy (Table 1). These medications were categorized according to groups with a defined pharmacological indication (Figure 1).

**Antimicrobial Agents (antiparasitic, antifungal, anthelmintic, and antiviral drugs)**

Several antimicrobial drugs in this class act by disrupting membranes, interfering with iron metabolism, or generating oxidative stress. These antimicrobial agents, despite exhibiting distinct mechanisms of action, frequently demonstrate synergistic activity when combined with antibiotics. Moreover, the majority of these compounds possess anti-inflammatory properties and exhibit immunomodulatory effects.

- Ciclopirox (antifungal): Functions through iron chelation and inhibition of LPS (lipopolysaccharide) synthesis, with broad activity against Gram-negative bacteria.
- Pentamidine (antiparasitic): An antibiotic sensitizer that interacts with LPS and increases membrane permeability, with potential for inhaled delivery formulations.
- Niclosamide (anthelmintic): Induces oxidative stress and inhibits ATP production; synergistic with polymyxins.
- Zidovudine: Demonstrates inhibition of plasmid transfer and bacterial DNA synthesis; exhibits synergy with several conventional antibiotics.

### 3.1. Ciclopirox

Ciclopirox, a topical antifungal medication, demonstrates direct antibacterial activity against clinical isolates of *A. baumannii*, *E. coli*, *K. pneumoniae*, and *P. aeruginosa*, with MIC values of 5–15 µg/mL (independent of bacterial sensitivity, with slightly lower sensitivity toward *P. aeruginosa* in some isolates) [17]. Its mechanism involves LPS synthesis and galactose metabolism, two pathways crucial for virulence. Another key mechanism of ciclopirox is iron chelation [17], a critical factor for bacterial virulence and host defense [18]. The drug may also act against biofilms, virulence factors, and synergistically with polymyxin B against Gram-negative bacteria, including resistant strains (*E. coli*, *A. baumannii*, *and P. aeruginosa*) [19]. In addition to its antibacterial effects, ciclopirox exhibits immunomodulatory properties [20], including inhibition of NLRP3 inflammasome activation and antioxidant activity [21]. Moreover, it inhibits the mammalian target of rapamycin (mTOR) [22], potentially activating autophagy (including in immune cells), which plays a crucial role in host defense against pathogens [23]. The high safety profile of ciclopirox (currently under clinical trial for oral administration, NCT05647343), the absence of reported resistance in antifungal treatments [24], its diverse potential sites of action, and host-directed potential (immunomodulatory) effects make it an excellent candidate for treating resistant Gram-negative bacteria. It is worth noting that ciclopirox has a short serum half-life, making it potentially not relevant for clinical settings. However, a potential solution is the use of a pro-drug (fosciclopirox), currently under clinical trials (NCT04956042). The efficacy of the pro-drug has been demonstrated in mice at a dose of 470 mg/kg, significantly higher than the parenteral doses of ciclopirox given to mice (ciclopirox LD50 in mice is 83–172 mg/kg, and doses given as treatment for various indications in this animal model are 1.25–25 mg/kg) [24,25,26,27,28,29].

### 3.2. Pentamidine

Pentamidine is used to treat parasitic infections, such as African trypanosomiasis and leishmaniasis, as well as for the prevention and treatment of pneumocystis pneumonia in immunocompromised patients [30]. Although direct antibacterial activity has been rarely reported, pentamidine has been proposed as a sensitizer (i.e., adjuvant) to conventional antibiotics, including those to which resistance has developed [30].

Synergistic effects were demonstrated against *E. coli* when combined with antibiotics primarily used against Gram-positive bacteria, such as vancomycin, novobiocin, and erythromycin. Pentamidine also showed adjuvant activity against a broad range of Gram-negative bacteria, including polymyxin-resistant strains, in in vivo models (mouse and *Galleria mellonella*). For example, synergy was observed in combination with novobiocin against *A. baumannii* [31]. In addition, in vitro synergy was demonstrated with other antibiotics such as linezolid, tigecycline, and doripenem against various clinical isolates of *E. coli*, *K. pneumoniae*, and *E. cloacae*. The combination with linezolid also led to in vivo synergy in *G. mellonella* [32].

The drug acts, among other mechanisms, by interacting with LPS, thereby compromising the integrity of the outer membrane, which is required to prevent antibiotic penetration into the bacterial cell. A recent screen combining pentamidine with 170 clinically approved antibiotics revealed promising combinations with minocycline, linezolid, valnemulin (a veterinary antibiotic), and nadifloxacin, including against resistant Gram-negative bacteria (MDR). In this study, it was suggested that hydrophobicity, rigidity, partial charge, and surface rugosity were key factors affecting sensitization via a cooperative membrane damage mechanism in which LPS and phospholipids were identified as sites of synergy. Specifically, the combination with linezolid slowed the development of resistance in vitro, alongside potent in vivo activity [33]. In a single study, combining pentamidine with the non-antibiotic drug auranofin led to synergistic in vitro effects against resistant Gram-negative bacteria; the MIC of auranofin was reduced by up to three orders of magnitude when combined with sub-MIC levels of pentamidine [34]. In preclinical studies examining chemical derivatives of pentamidine, compounds that are more effective were identified against a wide spectrum of Gram-negative bacteria, and in combination with antibiotics intended for Gram-positive pathogens, including in in vivo models. Some of these derivatives also showed improved safety profiles (e.g., reduced cytotoxicity [35,36]).

In addition to its antibacterial (adjuvant) effects, pentamidine exhibits anti-inflammatory effects, which may also possess beneficial effects (decreased morbidity and mortality) during bacterial infections. For instance, in a mouse model where LPS was injected, pentamidine treatment reduced serum levels of pro-inflammatory cytokines TNFα and IL-6, as well as TNFα in lung tissue, resulting in less tissue damage and significantly improved survival [37]. Anti-inflammatory effects were also demonstrated in a mouse model of colitis, showing reduced levels of PGE2, TNF-α, IL-1β, and decreased macrophage infiltration, potentially via inhibition of MD2, a TLR4 co-receptor [38]. The drug also reduced TNF-α levels and, to a lesser extent, IL-6 in rat serum following LPS injection, and significantly decreased edema severity in a carrageenan-induced paw inflammation model [39].

In mice infected with *C. difficile*, pentamidine led to a strong anti-inflammatory effect, evidenced by reduced levels of various pro-inflammatory cytokines (IL-1β, IL-18, IL-6, GM-CSF, TNFα, IL-17, IL-23, and IL-2), increased levels of IL-22 (associated with regenerative and antimicrobial functions) [40], and decreased neutrophil infiltration into the intestine [41]. The proposed mechanism in this case involves inhibition of S100B, a pro-inflammatory cytokine that acts upstream and activates multiple intracellular inflammatory pathways, such as RAGE, NF-κB, STAT3, and PI3K [41]. Further evidence suggests the inhibition of pro-inflammatory chemokine production in whole blood [42] and reduced production of pro-inflammatory cytokines (IL-6, TNF-α, and IL-1β) in alveolar macrophages stimulated with LPS at doses deemed clinically relevant [43].

### 3.3. Niclosamide

The anthelmintic drug niclosamide has emerged as a promising candidate for antibacterial therapy, particularly against resistant Gram-negative pathogens. Recent studies have demonstrated its multifaceted antibacterial effects, including quorum sensing-inhibition in *P. aeruginosa*, conferring protection in *G. mellonella* models [44], direct antibacterial activity against Gram-negative bacteria, including Carbapenemase-producing Enterobacteriaceae (CPE), with MIC values of 15 μg/mL for *K. pneumoniae* and *E. coli* isolates [45], and synergistic effects with polymyxins [46,47]. The drug’s mechanisms of action involve oxidative damage induction, ATP production disruption [48], and inhibition of bacterial surface motility and virulence factor production [44]. Notably, niclosamide exhibits immunomodulatory properties, suppressing pro-inflammatory cytokine secretion and NF-κB pathway activation in various models [49,50,51]. While poor oral bioavailability presents a challenge, ongoing research into pulmonary and intranasal formulations aims to overcome this limitation [52]. Two primary resistance mechanisms have been identified: efflux pump-mediated expulsion and nitro reduction, prompting investigations into combination therapies with efflux inhibitors or nitro-prodrug antibiotics [48]. The broad-spectrum activity of niclosamide, coupled with its ability to enhance the efficacy of existing antibiotics and modulate host immune responses, positions it as a valuable candidate for further clinical investigation in treating resistant bacterial infections.

### 3.4. Tavaborole

Tavaborole is a topical antifungal drug used topically, that has recently been identified as having antibacterial potential. This drug has demonstrated efficacy against *P. aeruginosa* and other Gram-negative bacteria, including *A. baumannii* and *B.* [53]. Additionally, Tavaborole exhibits an adjuvant effect when combined with aminoglycosides, including in a murine peritonitis model, while maintaining low toxicity both in vitro and in vivo [54].

### 3.5. Zidovudine

Known also as Azidothymidine (hereinafter: “AZT”). AZT is an antiretroviral drug used for the prevention and treatment of HIV/AIDS [55]. The antibacterial activity of AZT was demonstrated in vitro over 35 years ago against Gram-negative bacteria, including Enterobacteriaceae such as *E. coli*, *S. typhimurium*, *K. pneumoniae*, *S. flexneri*, and *E. aerogenes*, through a mechanism involving the inhibition of DNA synthesis. The AZT-triphosphate metabolite acts as a DNA chain terminator. It was also shown that AZT is a substrate of the bacterial enzyme thymidine kinase, and mutations in this enzyme are a known mechanism of bacterial resistance to AZT [56,57]. Another mode of action was discovered through a screen of 1200 FDA-approved drugs in *E. coli* and *K. pneumoniae*, which showed that AZT prevents the transfer of plasmids containing extended-spectrum β-lactamases (ESBLs) or carbapenemases [58]. In recent years, significant emphasis has been placed on combinations of antibiotics with AZT (where AZT serves as an adjuvant). It has been demonstrated that the AZT-meropenem combination is effective against multidrug-resistant (MDR) strains of *K. pneumoniae* producing carbapenemase in vitro and in vivo in *G. mellonella* larvae. Specifically, unlike AZT, meropenem was completely inactive both in vitro and in larvae (in vivo), and only the combination of AZT with meropenem showed efficacy (a 30–40% improvement in survival rates and a significant decrease in bacterial burden) [59]. Antibacterial synergy against MDR *K. pneumoniae* was also observed with the combination of AZT and polymyxin B both in vitro and in vivo (in mice), and it was suggested that the combination could reduce the development of resistance to polymyxins [60]. The potential of AZT-colistin combination was demonstrated in vivo in another study (mouse peritoneal infection model) [61], and in *K. pneumoniae* strains isolated from patients [62]. The drug also showed in vitro synergistic activity against colistin-resistant *E. coli* [63], and dramatically reduced resistance to tigecycline, including in vivo (in *G. mellonella* and mice) [64]. A combination with gentamicin against *E. coli* also demonstrated synergy [65]. Moreover, combinations with tigecycline or colistin showed in vitro and in vivo (mouse) efficacy against *E. coli* strains harboring both tet(X) and mcr-1 resistance genes [66]. Another antibiotic with which AZT demonstrated synergy against Gram-negative bacteria (*K. pneumoniae*, *E. coli*, *E. cloacae*), including in *G. mellonella* larvae, is fosfomycin [67]. AZT’s impressive synergistic potential has also been demonstrated in high-throughput screens. A screen of 2000 small molecules suggested combinations of antibiotics (e.g., trimethoprim or sulfamethiazole) that, when combined with AZT, showed efficacy against *E. coli* and *K. pneumoniae*, including resistant strains [68]. In another screen of 1163 FDA-approved drugs, AZT emerged as the most promising candidate for combination therapy with tigecycline against carbapenem-resistant Enterobacteriaceae (CRE, *E. coli*) [69].

**Anti-inflammatory agents (non-steroidal anti-inflammatory drugs and anti-rheumatic drugs)**

### 3.6. Non-Steroidal Anti-Inflammatory Drugs

Non-steroidal anti-inflammatory drugs (NSAIDs) such as Aspirin, ibuprofen, and diclofenac demonstrate modest direct antibacterial activity, often accompanied by the inhibition of biofilm formation, suppression of bacterial motility, and synergy with antibiotics. Their known anti-inflammatory properties further support host-directed benefits during infection.

- Diclofenac: Inhibits bacterial DNA synthesis and shows synergistic interactions with ciprofloxacin. Its achievable clinical dosing makes diclofenac a candidate for adjunctive therapy.
- Acetylsalicylic acid (Aspirin): Demonstrates synergy with colistin, including reversal of resistance in MDR Enterobacteriaceae. Salicylate inhibits biofilm formation and motility in *E. coli* and *Pseudomonas*, with enhanced effects when combined with EDTA. Host-directed actions include Aspirin-triggered resolvins that accelerate bacterial clearance in murine pneumonia models, with additive effects alongside ciprofloxacin.
- Ibuprofen: Exhibits MICs in the low µg/mL range against *E. coli*, inhibits biofilm maturation of *Pseudomonas*, and reduces adherence to urothelial cells by decreasing fimbriae production. In cystic fibrosis models, ibuprofen delays *Pseudomonas* biofilm development and improves survival in infected mice.

#### 3.6.1. Diclofenac

Diclofenac has demonstrated significant antibacterial activity against a wide range of Gram-negative bacteria in vitro. The effective antibacterial concentration of diclofenac varies considerably, even among isolates of the same bacterial species [70]. Evaluation of the antibacterial effect of diclofenac against clinical isolates of *Escherichia coli* from hospital-acquired urinary tract infections demonstrated efficacy against a substantial proportion of the isolates, with a minimum inhibitory concentration (MIC) of 2.5 µg/mL. Specifically, 55% of the isolates were susceptible, 15% exhibited intermediate susceptibility, and 33% were resistant. Furthermore, in vivo studies showed significant protective effects, with a mortality rate of 60% in untreated control mice compared to minimal mortality in treated mice across all administered doses (15–60 mg/kg). This protection was associated with a reduction in bacterial load in various organs [71]. The in vivo efficacy of diclofenac has also been demonstrated against *Salmonella enterica* serovar Typhimurium, with a proposed mechanism involving inhibition of DNA synthesis [72]. Beneficial effects, in terms of reduced histopathological damage and bacterial burden in tissues, were observed in a murine listeriosis model at clinically relevant doses (2.5 mg/kg/day), suggesting both direct antibacterial activity and a host-directed therapy (HDT) mechanism [73]. Additionally, diclofenac exhibited significant antibacterial activity against Salmonella in vitro and in vivo in a murine model. Notably, improved or synergistic effects were observed in mice when combined with streptomycin [74,75]. In vivo synergy against *S. Typhimurium* was also reported when diclofenac was combined with trifluoperazine, an antipsychotic drug that is not classified as an antibiotic [76]. More recently, a synergistic effect between diclofenac and fluoroquinolones (ciprofloxacin or levofloxacin) was demonstrated in vitro against extensively drug-resistant (XDR) *A. baumannii* [77]. At the mechanistic level, studies on *P. aeruginosa* have indicated that diclofenac, at sub-antibacterial concentrations, may inhibit the synthesis of virulence factors by interfering with biofilm formation and quorum-sensing regulation [78]. An additional potential advantage of diclofenac lies in its anti-inflammatory properties, characteristic of NSAIDs. This may be particularly beneficial when administered alongside antibiotics, even in the absence of a direct synergistic effect.

#### 3.6.2. Acetylsalicylic Acid (Aspirin)

Acetylsalicylic acid is an established anti-inflammatory drug used for analgesia, fever reduction, and anticoagulation [79]. Approximately 30 years ago, Ohsuka and colleagues demonstrated that Aspirin exhibits antibacterial activity against Gram-negative bacteria (*E. coli*, *P. aeruginosa*, and *S. Typhimurium*) with a minimum inhibitory concentration (MIC) of 10 µg/mL for all three species. The proposed mechanism of action involves disruption of the bacterial membrane. Furthermore, a synergistic effect was observed when Aspirin was combined at sub-inhibitory concentrations with novobiocin or nalidixic acid [80].

The combination of Aspirin or its active metabolite, salicylate, with colistin led to a synergistic effect and reversal of colistin resistance in multidrug-resistant (MDR) Gram-negative bacteria, including *E. coli*, *P. aeruginosa*, *K. pneumoniae*, and other Enterobacteriaceae [81]. Additionally, Aspirin exhibited antibacterial activity against *E. coli* and *P. aeruginosa* with MIC values ranging from 1.2 to 2.7 µg/mL, while also inhibiting biofilm formation by these bacteria. The biofilm inhibitory effect was enhanced when combined with EDTA [82]. Salicylate has been shown to suppress *E. coli* biofilm formation through the upregulation of the transcriptional regulator marA [83].

In addition, various antibacterial effects of salicylate against Gram-negative bacteria have been reported, including inhibition of motility in *B. cepacia*, *E. coli*, *P. aeruginosa*, and other species (with clinical relevance for *E. coli*). Furthermore, salicylate exhibited anti-virulence effects in *P. aeruginosa* and *K. pneumoniae*, as well as anti-metabolic effects in *E. coli* at concentrations exceeding clinically relevant plasma levels [84].

Aspirin’s role as a host-directed therapy (HDT) is well established, given its anti-inflammatory properties. In this context, a protective effect was demonstrated in a self-resolving murine pneumonia model following treatment with Aspirin-triggered Resolvin D1 (AT-RvD1), a lipid mediator generated both endogenously and upon Aspirin administration. This effect was associated with accelerated bacterial clearance of *E. coli* and *P. aeruginosa* from organs, enhanced phagocytosis of these bacteria, and increased neutrophil clearance from the lungs. Notably, these effects were additive when combined with ciprofloxacin [85].

#### 3.6.3. Ibuprofen

Ibuprofen is a widely used NSAID indicated for pain relief, fever reduction, and inflammation treatment [86]. The drug exhibits antibacterial activity against Gram-negative bacteria, such as *E. coli*, with reported minimum inhibitory concentration (MIC) values ranging from 1.25 to 5 µg/mL [87].

Ibuprofen also possesses anti-biofilm activity against Gram-negative bacteria, including *P. aeruginosa*, at clinically relevant plasma concentrations. This property suggests its potential as an adjunctive therapy in combination with antibiotics [88]. Supporting this notion, the combination of ibuprofen with ciprofloxacin demonstrated anti-virulence effects, including a significant reduction in biofilm formation, as well as impairment of bacterial motility (both swimming and swarming) and hemolytic activity in *P. aeruginosa* [89].

Furthermore, ibuprofen at low concentrations (2 µg/mL) was found to reduce the adherence of *E. coli* to urothelial epithelial cells. This effect was attributed to decreased fimbria production, key surface appendages, responsible for bacterial adhesion, as well as alterations in bacterial surface hydrophobicity [84]. Weak antibacterial activity was also observed against Gram-negative bacteria commonly found in cystic fibrosis (CF) patients, including *P. aeruginosa* and various Burkholderia species (*B. cenocepacia*, *B. multivorans*, and *B. cepacia*), at concentrations of 50–100 µg/mL. The proposed mechanism involves intracellular ATP depletion, even under physiologically relevant conditions such as sputum-like environments. Additionally, ibuprofen was shown to delay *P. aeruginosa* biofilm maturation [90]. In a murine model of pulmonary *P. aeruginosa* infection, oral administration of ibuprofen resulted in a 10-fold reduction in bacterial burden in the lungs and spleen. This effect was accompanied by significant clinical improvements and, most importantly, markedly increased survival rates (92% in the treated group vs. 57% in the untreated group, three days post-infection) [90].

### 3.7. Auranofin

Auranofin (AF) is a gold-containing compound that has been used for over 40 years to treat rheumatoid arthritis (RA). The drug exerts anti-inflammatory effects, such as inhibition of NF-κB activation, as well as antioxidant activity through the inhibition of thioredoxin reductase. However, the precise mechanism responsible for its therapeutic benefit in RA remains unclear. The clinical approval and increasing use of biologic therapies (e.g., anti-TNF and IL-1 receptor antagonist agents) have led to a decline in AF usage. Nevertheless, its potential for drug repurposing, particularly for cancer and antimicrobial applications, has renewed interest in this drug [91]. According to the literature, AF displays antibacterial activity against *E. coli* (MIC = 4 µg/mL) and *P. aeruginosa* (MIC = 16 µg/L, as well as carbapenem-resistant *A. baumannii* (MIC = 13.6 µg/mL) [92]. Despite its limited efficacy against Gram-negative bacteria as a monotherapy, synergistic antibacterial effects have been observed when AF is combined with polymyxin B or colistin, resulting in a dramatic reduction in MIC values against *E. coli*, *P. aeruginosa*, *A. baumannii*, *Klebsiella pneumoniae*, and *S. typhimurium*, including multidrug-resistant (MDR) strains [93,94,95,96]. In combination with pentamidine, AF demonstrated a synergistic effect against a broad spectrum of Gram-negative bacteria, including antibiotic-resistant strains of *A. baumannii*, *K. pneumoniae*, and *E. coli.* This combination led to a ≥1024-fold reduction in the MIC of AF, attributed to membrane-disruptive effects and increased intracellular accumulation of AF [34]. The study also showed that the AF/pentamidine combination delayed the development of AF resistance in *K. pneumoniae* when compared to the AF/colistin combination. In addition to inhibiting thioredoxin reductase [95], AF has specific adjuvant-related effects on certain bacterial species, including the inhibition of metallo-β-lactamases (MBLs) and mobilized colistin resistance (MCR) enzymes. These effects are partially mediated through the displacement of zinc (Zn^2+^) from the active sites of these enzymes [97]. It is essential to acknowledge several limitations associated with AF, including low oral bioavailability (~25% of the administered dose is absorbed) and subtherapeutic plasma concentrations for antibacterial activity [98]. Therefore, its use against Gram-negative bacteria may be limited to combination therapies with antibiotics or other antibacterial medical countermeasures.

**Cardiovascular and Metabolic Drugs**

The drugs listed below exhibit antibacterial activity in combination with antibiotics and are mainly characterized by immunomodulatory properties.

- Amlodipine: Inhibits efflux pumps and reverses resistance in vitro; however, pharmacological limitations prevent its use in acute sepsis.
- Metformin: Reduces the transcription of efflux pump genes; has favorable safety but limited direct antimicrobial potency.
- Statins: Show antibacterial effects and immunomodulation in vitro, possibly through altered membrane function and cholesterol biosynthesis pathways.

### 3.8. Amlodipine

Amlodipine is a calcium channel blocker widely used for the treatment of hypertension [99]. This drug has demonstrated synergistic effects when combined with several classes of antibiotics. For example, synergy with imipenem was observed in clinical isolates of *A. baumannii*, including drug-resistant strains, 50% of 42 patient-derived isolates showed synergism, and 25% exhibited additive effects [100]. In resistant *A. baumannii*, the synergistic interaction with imipenem is thought to result from the inhibition of the AdeABC efflux pump [101]. In vitro synergy was also demonstrated when amlodipine was combined with polymyxin B against carbapenem-resistant *A. baumannii* [102]. Synergistic effects have also been reported in combination with the aminoglycoside streptomycin, both in vitro against Gram-negative bacteria (*Shigella dysenteriae*, *S. typhimurium*, and *Vibrio cholerae*) and in vivo in a murine *S. typhimurium* infection model, where a significant reduction in bacterial burden was observed across organs [103]. Furthermore, amlodipine exhibited in vitro synergistic activity with levofloxacin by inhibiting biofilm formation by *P. aeruginosa* [104]. Additive activity has also been reported in vitro when combined with tetracycline antibiotics or with carvedilol, an antihypertensive agent without inherent antibacterial activity [105]. An additional benefit of combining amlodipine with certain antibiotics, such as peptidomimetic β-lactams (e.g., cephalexin), is enhanced oral bioavailability of the antibiotic due to drug–drug interaction [106]. The intrinsic antibacterial activity of amlodipine is limited, with minimum inhibitory concentrations (MICs) ranging from 50 to 200 µg/mL against several Gram-negative pathogens, including *E. coli*, *Klebsiella* spp., and *P. aeruginosa*. Thus, its therapeutic relevance likely lies in its role as an antibiotic adjuvant [107]. Notably, in vivo studies using a murine model of salmonellosis demonstrated that the observed antibacterial effects occurred at clinically relevant doses of amlodipine [108]. However, in the context of bacteremia with severe sepsis or septic shock, effective antibacterial therapy is defined as treatment that reduces the duration of hypotension [109]. Therefore, further investigation is warranted to determine specific clinical scenarios where combining amlodipine, an antihypertensive agent may offer therapeutic benefit.

In addition to its potential antibacterial properties, amlodipine has been reported to exert anti-inflammatory effects. In an in vitro model of AGE-LDL-induced endothelial dysfunction, it demonstrated protective effects by reducing inflammatory damage (via decreased expression of MCP-1 and VCAM-1) and oxidative stress [110]. Another anti-inflammatory mechanism involves suppression of plasminogen receptor expression on macrophages. In in vivo murine models, amlodipine reduced thioglycolate-induced peritoneal macrophage recruitment by 60% at a dose of 3 mg/kg, without affecting systemic blood pressure [111,112].

### 3.9. Metformin

Metformin is a biguanide-class antidiabetic drug, primarily used in the treatment of type 2 diabetes mellitus [113]. Recent studies have demonstrated that metformin can restore sensitivity to tetracyclines in multidrug-resistant (MDR) bacterial strains, including Gram-negative pathogens such as *E. coli.* This effect is mediated through intracellular accumulation of tetracycline, likely via inhibition of tet(A) gene transcription, along with immunomodulatory activity. The therapeutic potential has been validated in in vivo infection models using *G. mellonella* and mice [114]. Metformin has also shown synergistic antibacterial activity when combined with various antibiotics, including levofloxacin, chloramphenicol, ampicillin, rifampicin, and doxycycline, while maintaining a favorable cytotoxicity profile in mammalian cells [115]. In a murine model of *E. coli*-induced pneumonia, metformin enhanced macrophage-mediated bacterial phagocytosis through AMPK activation [116]. The drug’s immunomodulatory effects include suppression of pro-inflammatory cytokine production in bacteria-host cell co-culture systems [114] and enhancement of innate immune tolerance to pathogens via activation of the p38 MAPK pathway [117]. Additionally, direct anti-inflammatory effects have been reported following LPS exposure, attributed to AMPK signaling activation (mentioned above in context of macrophages) also in epithelial cells [118] and induction of fibroblast growth factor-21 (FGF21) in the liver [119]. A notable secondary mechanism involves metformin-mediated reduction of glucose concentrations in the airway surface liquid, thereby restricting nutrient availability for invading bacteria and contributing to airway protection against bacterial infections [120]. Furthermore, metformin has been shown to protect against LPS-induced cardiac injury, suggesting potential benefits during systemic infections caused by Gram-negative bacteria [121]. In summary, metformin exerts two primary antibacterial effects: (1) restoration of bacterial sensitivity to tetracyclines and other antibiotics, particularly in resistant strains, and (2) indirect antibacterial activity through immunomodulatory mechanisms.

### 3.10. Statins

The direct antibacterial activity of statins, drugs used to treat hyperlipidemia, against Gram-negative bacteria is limited [122], if present at all [123]. Specifically, atorvastatin, a member of the statin family, has MIC values of approximately 15 µg/mL, 15 µg/mL, and 25 µg/mL against *A. baumannii* (ATCC 17978), *Enterobacter aerogenes* (ATCC 29751), and *E. coli* (ATCC 35218), respectively. Atorvastatin maintained its activity against clinical strains (approximately 20 µg/mL against *A. baumannii* and *E. aerogenes*). Simvastatin also exhibits limited activity against these two clinical strains (approximately 30 µg/mL) [124]. In other strains (ATCC), no efficacy of atorvastatin was demonstrated against *A. baumannii* [125]. Limited effects (MIC ≥ 50 µg/mL) of various statins have been demonstrated against other Gram-negative bacteria [126]. Despite their limited antibacterial effect, statins have significant adjuvant potential due to their anti-inflammatory [127] and immunomodulatory effects [128]. When administered in combination with imipenem (*K. pneumoniae* in mice), a marked improvement in survival rates was achieved due to a clear immunomodulatory effect (without affecting the bacterial load in the blood and lungs). This included significant reductions in both pro-inflammatory cytokines (TNFα and IL-6) and the infiltration of inflammatory cells (macrophages and neutrophils) into the lung tissue, alongside improvements in histopathological damage scores [129].

**Gastrointestinal Agents**

- Loperamide: Disrupts bacterial membranes and increases permeability. It could have antagonistic effects with certain antibiotics; despite in vitro potency, pharmacokinetics limit systemic use.
- Bismuth: Specifically eradicates MDR P. aeruginosa in combination with antibiotics.

### 3.11. Loperamide

Loperamide is an opioid drug (μ-opioid receptor agonist) commonly used as an anti-diarrheal agent (marketed as Imodium) [130]. The compound has demonstrated in vitro and in vivo (murine model) synergistic effects when combined with colistin against multidrug-resistant Enterobacterales (*E. coli*, *K. pneumoniae*), particularly strains harboring plasmid-mediated mcr-1 genes. The proposed mechanism of action includes mechanical disruption of the bacterial membrane and direct binding to the MCR-1 protein, resulting in its inhibition [131].

Additionally, the combination of loperamide with tetracyclines, such as minocycline, exhibited in vitro synergistic effects against *P. aeruginosa* and *E. coli*, including multidrug-resistant (MDR) strains. In *E. coli*, loperamide was shown to permeabilize the outer membrane, enhancing tetracycline uptake. Synergistic in vitro activity was also observed when loperamide was combined with cephalosporins, though this effect appeared to be antibiotic-specific, as it was not replicated with other β-lactams targeting the bacterial cell wall. Similarly, synergy was reported with polymyxin B, likely via increased membrane permeability. Conversely, combinations with aminoglycosides yielded antagonistic effects, underscoring the need for careful evaluation of antibiotic pairings involving loperamide [132].

Beyond its role as an adjuvant, loperamide exhibits immunomodulatory effects, including suppression of TNF-α levels in a *Mycobacterium*-macrophage co-culture system. Although its intrinsic antimicrobial activity is limited (MIC = 100–150 µg/mL), studies have shown some efficacy against mycobacteria, partly through the induction of antimicrobial peptides [133,134]. It should be noted that loperamide is poorly absorbed following oral administration [135]. Therefore, in therapeutic regimens involving loperamide-antibiotic combinations, parenteral administration of loperamide would be required. Currently, no clinically approved parenteral formulation exists.

### 3.12. Bismuth 

Bismuth compounds have historically been utilized in the treatment of infectious diseases such as syphilis, malaria, and wound infections. Currently, they are primarily employed in the management of *Helicobacter pylori* (*H. pylori*)-associated peptic ulcers [136,137,138]. Notably, the combination of bismuth with antibiotics constitutes an effective therapeutic strategy for eradicating *H. pylori*, including antibiotic-resistant strains [139]. Despite its clinical efficacy, the use of bismuth in *H. pylori* treatment remains largely empirical. Recent evidence suggests that bismuth compounds hold potential for drug repurposing, particularly in addressing metallo-β-lactamase-producing and tigecycline-resistant bacterial infections, by inhibiting metallo-β-lactamases and tetracycline-inactivating enzymes [140,141]. Recently, it was reported that bismuth-based compounds, including bismuth subsalicylate and colloidal bismuth subcitrate, in combination with various classes of antibiotics (e.g., tetracyclines, macrolides, quinolones, rifamycins), effectively eradicate multidrug-resistant *P. aeruginosa* without promoting the development of antibiotic resistance. Mechanistically, bismuth disrupts iron homeostasis by binding to *P. aeruginosa* siderophores, thereby interfering with bacterial iron acquisition. Intracellularly, bismuth impairs the electron transport chain (ETC), dissipates the proton motive force (PMF), and inhibits efflux pump activity by targeting iron–sulfur cluster-containing enzymes, including respiratory complexes. These disruptions lead to increased intracellular antibiotic accumulation, thereby enhancing antibacterial efficacy. The combination therapy exhibits potent antibacterial activity and low toxicity in an ex vivo bacteremia model, and significantly improves survival rates in murine lung infection models. These findings underscore the potential of repurposing bismuth-based drugs as antibiotic adjuvants to combat MDR *P. aeruginosa* infections in clinical settings [142].

**Psychotrophic medications (antipsychotics, antidepressants and anxiolytics)**

- Fluspirilene: Show limited but notable activity; more research required.
- SSRIs: Manifest antibacterial activity and synergy with aminoglycosides and fluoroquinolones; sertraline and fluoxetine have been most studied.
- Anxiolytics (benzodiazepines): Some members promote plasmid DNA cleavage, interfering with resistance gene transfer.

### 3.13. Fluspirilene

Fluspirilene, an older-generation antipsychotic drug, has been identified in comprehensive screenings of FDA-approved (or inclusive of approved) drug libraries as a potential antibiotic resistance breaker (ARB), and in certain instances, a direct antibacterial agent [143]. This compound has demonstrated the ability to restore colistin sensitivity in several resistant bacterial strains, leading to a notable enhancement in antibiotic activity against *A. baumannii* (4-fold increase), *E. coli* (>128-fold increase), and *P. aeruginosa*. Moreover, fluspirilene exhibits a direct, albeit limited, antibacterial effect against colistin-resistant *A. baumannii* (MIC = 20 µg/mL) [143]. Synergistic effects with colistin were confirmed in another study, revealing a 30-fold reduction in the IC50 of the antibiotic against an *A. baumannii* strain resistant to 25 first-line antibiotics typically used against Gram-negative bacteria [144].

Beyond its potential as an ARB, fluspirilene mediates indirect, host-directed antibacterial effects within macrophages against intracellular Gram-negative bacteria such as *Mycobacterium tuberculosis* and *Salmonella enterica* serovar Typhimurium (including resistant strains). The antibacterial mechanism involves modulating autophagy and the lysosomal response, resulting in increased bacterial presence within autophago(lyso)somes, oxidative damage, and acidification of lysosomes [145].

### 3.14. Selective Serotonin Reuptake Inhibitors 

Selective serotonin reuptake inhibitors (SSRIs) are a class of antidepressant medications, some of which possess antibacterial properties, particularly in combination with other antibiotics, as detailed below. Sertraline and citalopram have demonstrated synergistic effects with polymyxin B against *P. aeruginosa*, *K. pneumoniae*, *E. coli*, and *A. baumannii* [146]. Additional synergy between sertraline and other antibiotics, including fluoroquinolones and gentamicin, has been observed against *P. aeruginosa* and *E. coli* [147]. Furthermore, sertraline has been shown to restore tetracycline sensitivity in *E. coli* by reversing resistance mediated by TetA (an efflux pump) [148]. Fluoxetine exhibits a synergistic effect against *P. aeruginosa* and *E. coli* when combined with gentamicin or erythromycin [149]. Synergy has also been observed when ciprofloxacin is combined with fluoxetine or paroxetine against *K. pneumoniae* and *A. baumannii* (including resistant strains), alongside direct but limited antibacterial activity (e.g., MIC = 32 µg/mL for fluoxetine against *E. coli*) [150]. In addition to their antibacterial potential, SSRIs possess anti-inflammatory activity, including inhibition of pro-inflammatory cytokine secretion and suppression of classical inflammatory elements such as NFκB, inflammasome, TLR4, and PPARγ. They also inhibit acid sphingomyelinase activity in epithelial and immune cells [151].

### 3.15. Benzodiazepines

Benzodiazepines are medications used, among other things, to treat anxiety and sleep disorders. A recent study indicates that a potent synergistic effect is achieved in vitro by combining clonazepam or diazepam with ciprofloxacin against resistant *K. pneumoniae*, *A. baumannii*, and *Enterobacter cloacae* [152]. Another study suggests that combining clonazepam with ciprofloxacin or sulfamethoxazole-trimethoprim leads to synergism through oxidative and hydrolytic cleavage of plasmids [153].

**Table 1:** Antibacterial Mechanisms of Action and Additional Properties of Repurposed Drugs.

Drug Name	Original Indication	Antibacterial Mechanism(s) of Action	Antibacterial Activity (In Vitro)	In Vivo Activity	Immune-Modulation/Antioxidant Effect	Anti-Virulence Effect
Ciclopirox	Antifungal (topical)	Iron chelation; inhibition of LPS synthesis, disruption of galactose metabolism	+	+	+	+
Pentamidine	Anti-parasitic	Adjuvant (LPS interaction; increased membrane permeability)	+(as an adjuvant)	+(as an adjuvant)	+	
Niclosamide	Anthelmintic	Oxidative damage; inhibition of ATP production	+	+	+	+
Tavaborole	Antifungal (topical)	Not specified	+	+(as an adjuvant)		
Zidovudine	Antiviral	Inhibition of DNA synthesis; inhibition of plasmid transfer	+	+(as an adjuvant)		
Diclofenac	NSAID	Inhibition of DNA synthesis	+	+	+	+
Acetylsalicylic acid (ASA)	NSAID	Membrane disruption	+	+	+	+
Ibuprofen	NSAID	Inhibition of ATP production	+	+	+	+
Auranofin	Anti-rheumatic	Inhibition of thioredoxin reductase (TrxR); inhibition of MBLs and MCR enzymes	+		+	
Amlodipine	Antihypertensive	Adjuvant (inhibition of efflux pumps)	+(as an adjuvant)	+(as an adjuvant)	+	
Metformin	Antidiabetic (T2DM)	Inhibition of efflux pump transcription	+(as an adjuvant)	+(as an adjuvant and through immune modulation)	+	
Statins	Antihyperlipidemic	Not specified	+	+(as an adjuvant)	+	
Loperamide	Antidiarrheal	Adjuvant (membrane disruption, increased permeability)	+		+	
Bismuth	Peptic ulcers and diarrhea	Adjuvant (inhibition of antibiotics-inactivating enzymes and efflux pumps)	+	+		
Fluspirilene	Antipsychotic	Adjuvant and host-directed effects	+(as an adjuvant)			
SSRIs	Antidepressants	Adjuvant	+(as an adjuvant)	+(as an adjuvant)	+	
Benzodiazepines	Anxiolytics	Adjuvant (plasmid cleavage)	+(as an adjuvant)			

Abbreviations: MBLs = metallo-β-lactamases; MCR = mobilized colistin resistance; NSAID = non-steroidal anti-inflammatory drug; SSRIs = selective serotonin reuptake inhibitors; T2DM = type 2 diabetes mellitus, TrxR = thioredoxin reductase.

**Anticancer and Cytotoxic Agents**

Several anticancer drugs exhibit antibacterial properties by inhibiting DNA replication or interfering with protein synthesis. Their cytotoxicity may limit therapeutic application, but they provide valuable scaffolds for development.

### 3.16. Anticancer Drugs

The repurposing of anticancer drugs for antibacterial therapy presents a significant challenge due to the adverse side effects commonly associated with these agents. Nevertheless, several compounds—selected from a relatively broad list proposed in the literature—have demonstrated promising antibacterial potential [154,155].

#### 3.16.1. *5-Fluorouracil*

5-Fluorouracil (5-FU), an antimetabolite, has been shown to inhibit growth and biofilm formation in *E. coli* (MIC = 10 µM) and *P. aeruginosa* (MIC = 0.5–1 µg/mL). In *P. aeruginosa*, 5-FU also suppressed quorum sensing and virulence gene expression [156,157]. Protective activity against *P. aeruginosa* was further demonstrated in a murine infection model following treatment with fluorocytosine, a prodrug of 5-FU [158]. Clinical studies have indicated that 5-FU, when used for catheter coating in critically ill patients, was both safe and effective in preventing Gram-negative bacterial infections and subsequent bacteremia, although these findings do not necessarily support systemic administration. Furthermore, synergistic antibacterial activity was observed when 5-FU was combined with β-lactam antibiotics [159].

#### 3.16.2. *Mitomycin C*

Mitomycin C (MMC) is a DNA crosslinking agent with demonstrated in vitro activity against *E. coli* and *P. aeruginosa*, with reported MIC50 values ranging from 0.2 to 15 µg/mL [160]. In the case of *A. baumannii*, MMC exhibited potent antibacterial activity, including efficacy against bacteria in the stationary phase and within biofilms. Furthermore, in vivo protection was demonstrated in the *G. mellonella* infection model [161]. Moreover, a combination therapy of MMC and bacteriophages resulted in a protective effect in vitro and in vivo (*G. mellonella*) against *K. pneumoniae* strains resistant to imipenem [162].

#### 3.16.3. *Hormonal Modulators*

The selective estrogen receptor modulators (SERMs, used for various estrogen-related diseases, including osteoporosis or treatment and risk reduction in breast cancers) raloxifene and tamoxifen have demonstrated antimicrobial properties. Raloxifene significantly reduced the virulence of *P. aeruginosa* in a *Caenorhabditis elegans* (*C. elegans*) infection model [163]. Tamoxifen enhanced neutrophils’ in vitro bactericidal activity against *E. coli* and *P. aeruginosa* [164]. Prophylactic administration of tamoxifen in murine models infected with multidrug-resistant strains of *A. baumannii*, *P. aeruginosa*, and *E. coli* resulted in a significant reduction in bacterial burden in blood and tissues, and markedly improved survival via an immunomodulatory mechanism [165]. In combination with polymyxin B, an antibiotic that was otherwise ineffective alone, SERMs, particularly toremifene, exhibited synergistic activity against extensively drug-resistant (XDR) pathogens, including *P. aeruginosa*, *K. pneumoniae*, and *A. baumannii* [166]. Beyond these antimicrobial effects, tamoxifen has also been proposed as a host-directed therapy (HDT) for tuberculosis. At sub-antibacterial doses, it modulated autophagy pathways—specifically lysosomal function—leading to enhanced lysosomal localization of *M. tuberculosis* both in vitro (in macrophages) and in vivo (in zebrafish larvae) [167]. Tamoxifen’s potentiation of innate immune responses has been demonstrated in macrophages, with noted antimicrobial effects against both intracellular pathogens and antibiotic-resistant bacteria. These effects appear to involve off-target mechanisms independent of estrogen receptor signaling, such as activation of the NRF2 pathway and caspase-1, implicating roles in immunometabolism and inflammatory regulation [168]. Clinically, low-dose tamoxifen administration has been associated with reduced serum C-reactive protein (CRP) levels in women, including in healthy individuals, further suggesting its potential as an immunomodulatory agent [169].

#### 3.16.4. *Gallium*

Gallium nitrate is clinically approved for the treatment of symptomatic hypercalcemia in cancer patients, primarily through inhibition of osteoclast-mediated bone resorption [170]. Gallium citrate, incorporating the Ga-67 isotope, is utilized in oncologic imaging [171]. Gallium acts as a non-reducible iron mimetic, disrupting bacterial iron metabolism [172]. Gallium citrate demonstrates broad-spectrum antimicrobial activity against a variety of Gram-negative bacteria, with reported minimum inhibitory concentrations (MICs) ranging from 1–5 µg/mL. Its efficacy extends to biofilm-associated bacteria and exhibits a low propensity for resistance development. Notably, it has shown in vivo effectiveness in infection models [172]. A combination of gallium nitrate and gallium protoporphyrin resulted in synergistic antimicrobial effects in vitro (including within macrophages and against biofilms) and in vivo, as evidenced by protection in *C. elegans* and reduced mortality in a murine pulmonary infection model. The mechanism involves disruption of iron and heme metabolism, coupled with induction of oxidative stress [173,174].

Gallium nitrate has also demonstrated efficacy against 20 polymyxin-resistant *K. pneumoniae* strains (MIC = 2–16 µg/mL), including in a *C. elegans* infection model, and showed synergism when combined with polymyxin B [175]. It has been proposed as an adjuvant (potentiator) to colistin against *K. pneumoniae*, both in vitro and in a murine pneumonia model, primarily via oxidative damage mechanisms [176]. Furthermore, gallium nitrate has shown in vitro activity (MIC = 2–80 µg/mL across 58 strains, including resistant isolates) and in vivo efficacy in the *G. mellonella* model at clinically relevant doses against *A. baumannii*. It also acts synergistically with colistin [177] and has been shown to inhibit biofilm formation by *A. baumannii* [178].

It should be noted that prolonged intravenous administration of gallium can be nephrotoxic, potentially limiting dosing and pulmonary tissue accumulation. To address this, a preclinical study investigated inhaled delivery of gallium, which demonstrated both efficacy and safety [179].

#### 3.16.5. *Tirapazamine*

Tirapazamine is a chemotherapeutic agent known for its selective cytotoxicity under hypoxic conditions, typically targeting tumor cells. In a recent high-throughput screen designed under conditions relevant to pulmonary infections in cystic fibrosis (CF) patients, tirapazamine was identified as having antibacterial activity against *P. aeruginosa*. This included both inhibition of biofilm formation and activity against pre-established biofilms [53]. The compound also demonstrated in vitro efficacy against *E. coli* and reduced bacterial burden in tissues while improving survival rates in a murine infection model. A proteomic analysis conducted as part of this study provided mechanistic insights into tirapazamine’s antibacterial effects, revealing differential expression of approximately 100 bacterial proteins, about half of which were upregulated and half downregulated [180]. As both of these studies were published recently, further research in this area is expected shortly.

Notably, tirapazamine’s in vitro antibacterial activity against *E. coli* was first reported nearly a decade ago. That earlier study demonstrated that the compound was effective under both anaerobic and aerobic conditions and retained activity against fluoroquinolone-resistant strains [181].

#### 3.16.6. *Mitoxantrone*

Mitoxantrone (MX) exhibits broad-spectrum antibacterial activity against multidrug-resistant (MDR) bacteria under physiologically relevant conditions (RPMI medium) and synergizes with colistin to combat MCR-positive pathogens. Notably, MX’s antibacterial efficacy was independent of key resistance determinants, including blaNDM, mcr, and tet(X4), which confer resistance to last-line antibiotics in Gram-negative bacteria. Mechanistically, MX enhances antibacterial activity by inducing membrane disruption, reactive oxygen species (ROS) generation, and DNA damage in *Salmonella enterica* serovar Enteritidis and *E. coli* strains. Scanning electron microscopy (SEM) revealed pore formation on bacterial membranes following MX treatment. Furthermore, supplementation with divalent cations (Mg^2+^, Ca^2+^)—which stabilize bacterial outer membranes—attenuated MX’s activity, supporting a membrane-targeting mechanism. Additionally, MX triggered intracellular Fe^2+^ accumulation, impairing lipopolysaccharide (LPS) modification and exacerbating oxidative stress through the Fenton reaction, culminating in bacterial cell death. Consistent with its anticancer mechanism of DNA intercalation and synthesis inhibition, MX induced significant bacterial DNA damage. Beyond direct antibacterial effects, MX modulates host immunity. Recent studies indicate that MX promotes macrophage recruitment, elevates proinflammatory cytokine expression, and enhances bacterial clearance in vivo.

Importantly, MX significantly potentiated the activity of colistin against mcr-positive bacteria. By disturbing iron homeostasis and downregulating mcr-1 expression, MX restored colistin’s efficacy, overcoming the lipid A modifications that typically confer colistin resistance. This synergistic effect offers a promising strategy to address the clinical challenge of colistin-resistant infections [182].

## 4. Natural Compounds, Vitamins, and Dietary Supplements

Phytochemicals are plant-derived compounds with diverse biological activities, including roles in plant defense against oxidative stress and pathogenic microorganisms. Accordingly, extensive literature supports their potential antibacterial effects [183]. In the following subsection, we highlight selected phytochemicals (as well as vitamins, nutrients and other dietary supplements), chosen from a broader list of candidates, that may serve as adjuncts or leads in antibacterial therapy. Importantly, due to their favorable safety profiles, these compounds warrant further investigation and potential inclusion in human treatment regimens, even in the absence of definitive clinical efficacy data or established antibacterial activity at physiologically relevant concentrations.

### 4.1. Curcumin

Curcumin, a polyphenolic compound derived from *Curcuma longa*, has been extensively studied for its antibacterial properties, particularly against Gram-negative bacteria. Its mechanisms of action include disruption of bacterial membranes and inhibition of virulence factor production and biofilm formation. In addition to its intrinsic antibacterial activity, curcumin has demonstrated synergistic effects when combined with various antibiotics, including polymyxin B, colistin, ciprofloxacin, and tetracycline, as well as with other bioactive compounds such as berberine and epigallocatechin gallate [184]. Notably, the combination of curcumin with polymyxins offers potential safety advantages. In preclinical studies, co-administration attenuated polymyxin-induced nephrotoxicity and neurotoxicity in both mouse and rat models [185]. Beyond its antimicrobial properties, curcumin is also recognized for its anti-inflammatory effects and excellent safety profile [186,187].

### 4.2. Berberine

Berberine is a phytochemical with limited intrinsic antibacterial activity [188]. However, its potential as an adjuvant in antimicrobial therapy is highly promising. While berberine alone does not exhibit significant efficacy against multidrug-resistant (MDR) *A. baumannii*, it has demonstrated strong synergistic effects when combined with various antibiotics. These combinations have been shown to restore bacterial susceptibility to agents such as tigecycline, sulbactam, meropenem, and ciprofloxacin, and have proven effective in vivo in neutropenic mouse models when used in combination with sulbactam [189]. At a concentration of 63.5 μg/mL, berberine also inhibited biofilm formation in clinical isolates of *K. pneumoniae* [190]. Moreover, when administered with imipenem against resistant *P. aeruginosa*, berberine restored antibiotic susceptibility through inhibition of the MexXY-OprM efflux system, resulting in a synergistic antibacterial effect [191]. In addition to its antimicrobial adjuvant properties, berberine exhibits anti-inflammatory and antioxidant activities, mediated in part by inhibition of NF-κB signaling and activation of AMPK [192,193].

### 4.3. Epigallocatechin Gallate

Epigallocatechin gallate (EGCG) is a polyphenolic compound primarily derived from dried tea leaves, particularly green tea [194]. Although its intrinsic antibacterial activity against Gram-negative bacteria is relatively limited, EGCG has demonstrated significant synergistic effects when combined with antibiotics. Notably, co-administration with gentamicin against multidrug-resistant (MDR) *E. coli* resulted in a marked reduction in the minimum inhibitory concentration (MIC) of EGCG from 1250 µg/mL to 156 µg/mL, while the MIC of gentamicin decreased from 32 µg/mL to 6.4 µg/mL [195]. In another study, EGCG restored susceptibility to aztreonam in MDR *P. aeruginosa*, significantly improving survival in the *G. mellonella* infection model compared to aztreonam monotherapy [196]. Additional evidence suggests that EGCG enhances *P. aeruginosa* susceptibility to chloramphenicol and tetracyclines by ≥4-fold, likely via inhibition of efflux pump activity [197]. Beyond its antibacterial potential, EGCG exhibits well-characterized anti-inflammatory and antioxidant properties, as extensively documented in the literature [198], including demonstrated efficacy in preclinical models of lung injury in mice and rats [199,200,201].

### 4.4. Quercetin

Quercetin is a plant-derived flavonoid that exhibits antibacterial activity against Gram-negative bacteria, including resistant strains. Its mechanisms of action include disruption of the bacterial membrane, inhibition of nucleic acid and protein synthesis, downregulation of virulence factor expression, inhibition of biofilm formation, and induction of mitochondrial dysfunction. The minimum inhibitory concentration (MIC) for *P. aeruginosa* has been reported as 20 µg/mL [202]. Furthermore, quercetin has been shown to inhibit carbapenemases and efflux pumps in carbapenem-resistant Gram-negative pathogens, with reported MIC values ranging from 16 to 256 µg/mL. In addition to its antibacterial effects, quercetin possesses potent anti-inflammatory and antioxidant properties and has demonstrated a favorable safety profile in murine models, with minimal adverse effects even at high doses [203,204].

### 4.5. Capsaicin

Capsaicin, a phytochemical derived from chili peppers (*Capsicum* spp.), has garnered significant interest for its potential antibacterial properties, particularly in the context of combating antimicrobial resistance. Its direct antibacterial activity has been demonstrated against a range of Gram-negative bacteria, with reported minimum inhibitory concentrations (MICs) of 64 µg/mL for *A. baumannii*, 5 µg/mL for *E. coli*, 0.6 µg/mL for *K. pneumoniae*, and 10 µg/mL for *P. aeruginosa* [205,206,207]. Importantly, capsaicin exhibited a synergistic effect when combined with colistin against colistin-resistant *A. baumannii*, reducing bacterial load in both organs and bloodstream in a murine infection model. This combination impaired biofilm formation and acted through mechanisms that include increased bacterial membrane permeability, disruption of ATP synthesis, and induction of oxidative stress [208].

### 4.6. Cranberry Proanthocyanidins

Plant-derived polyphenols extracted from cranberries (*Vaccinium macrocarpon*) have been attributed with a range of health-promoting properties, including antibacterial effects. Cranberry Proanthocyanidins (PACs) have been shown to enhance the efficacy of β-lactam antibiotics against resistant bacterial strains [209] and to prevent the development of antibiotic resistance, specifically to tetracycline, in *E. coli* and *P. aeruginosa*. Moreover, PACs demonstrated synergistic in vivo activity when combined with sulfamethoxazole (SMX) in *G. mellonella* and *Drosophila melanogaster* infection models targeting *P. aeruginosa*. Similarly, a combination of PACs and gentamicin yielded a significant synergistic effect, improving survival rates in *G. mellonella* from 20% to 70% compared to monotherapy [210]. The antibacterial mechanisms of PACs involve increased bacterial membrane permeability and inhibition of efflux pump activity [211]. Synergistic interactions were also observed between PACs and β-lactam antibiotics such as cefotaxime and ampicillin against clinical isolates of extended-spectrum β-lactamase (ESBL)-producing Enterobacteriaceae, including *E. coli*. In addition to direct antibacterial activity, PACs exhibit notable anti-virulence effects. For instance, they inhibit swarming motility and biofilm formation by *P. aeruginosa*. In contrast, no significant inhibition of growth or biofilm formation was observed in *E. coli* under similar conditions.

Further studies revealed that combining PACs with ciprofloxacin significantly impaired various virulence traits of *P. aeruginosa*, such as motility, biofilm production, and host cell invasion, phenotypes regulated by quorum sensing [212]. Notably, these anti-virulence effects were also evident following PACs monotherapy in *D. melanogaster* models [213], with in vivo biofilm inhibition confirmed in *G. mellonella* [213]. Additionally, co-administration of PACs and SMX reduced biofilm production in *E. coli*, *P. aeruginosa*, and *Proteus mirabilis* [211]. Regarding immunomodulatory potential, in vitro studies have shown that PACs interfere with the binding of lipopolysaccharides (LPS) to their receptors on human cells, preventing receptor internalization and leading to anti-inflammatory effects [214]. Furthermore, PACs inhibited the expression and activity of matrix metalloproteinases MMP-1 and MMP-9 in vitro [215]. Protective effects were also observed in macrophages and epithelial cells exposed to LPS and bacterial cell wall components [216]. The potent antioxidant properties of PACs further support their potential as protective agents in inflammatory settings [217].

### 4.7. Vitamin D

Although vitamin D lacks intrinsic antibacterial activity, it has shown promise as an adjuvant in the treatment of Gram-negative bacterial infections. Specifically, it has been demonstrated to restore and enhance the activity of aminoglycosides (amikacin, gentamicin, and kanamycin) against resistant strains of *E. coli*, *K. pneumoniae*, and *Shigella sonnei*. This effect is primarily attributed to the inhibition of aminoglycoside-N-acetyltransferase, an enzyme responsible for antibiotic inactivation [218]. Another proposed mechanism involves increased membrane permeability to aminoglycosides, mediated by the lipophilic interaction between the antibiotics and the vitamin D molecule [219]. In addition to its role as an antibiotic adjuvant, vitamin D has potential as a host-directed therapy (HDT), including applications against Gram-negative bacteria [220]. Mechanistically, its immunomodulatory functions are mediated through the induction of autophagy [221] and the upregulation of endogenous antimicrobial peptides, such as cathelicidin [222,223]. These findings support the potential utility of vitamin D as an adjunctive or supportive therapy in the management of resistant bacterial infections.

### 4.8. Vitamin C

Vitamin C exhibits a range of biological effects, including potent antioxidant properties, immunomodulatory functions—such as stimulation of immune cells and suppression of inflammatory responses and, to a lesser extent, direct antimicrobial activity. In vitro studies have demonstrated significant inhibitory effects of vitamin C against *P. aeruginosa*, with effective inhibition observed following exposure to a concentration of 0.31 µg/mL [224]. Beyond its direct antibacterial effects, vitamin C has been shown to enhance the activity of non-antibiotic compounds. For example, it potentiated the antibacterial effect of quercetin against *E. coli* [225], and synergized with desferrioxamine to inhibit a range of Gram-negative pathogens, including *E. coli*, *P. aeruginosa*, *K. pneumoniae*, and various Salmonella species [226]. These findings highlight the potential of vitamin C as a supportive therapeutic agent in antibacterial regimens, particularly in combination therapies targeting resistant Gram-negative bacteria.

### 4.9. Melatonin

Melatonin is an endogenously produced neurohormone secreted by the pineal gland, primarily involved in the regulation of circadian rhythms. Due to its well-established safety profile, ease of synthesis, and physiological role, melatonin is widely available over-the-counter (OTC) as a dietary supplement and is clinically used to manage sleep disorders and alleviate jet lag symptoms [227]. Beyond its chronobiological functions, melatonin has demonstrated antibacterial potential, with minimum inhibitory concentrations (MICs) of 31.25 µg/mL reported against carbapenem-resistant *A. baumannii* and *P. aeruginosa* [228]. Proposed bacteriostatic mechanisms include disrupting metal ion homeostasis within the cytoplasm, interfering with cell wall biosynthesis, modulation of bacterial cell division genes, and inhibiting key metabolic enzymes [229]. Specifically, in *Pasteurella multocida*, melatonin was shown to inhibit citrate synthase, thereby impairing citric acid production, leading to metabolic dysfunction and direct antimicrobial effects (MIC = 1.6–12.5 µg/mL). Administration of melatonin in infected murine models improved survival outcomes [230]. Melatonin has also exhibited synergistic effects when combined with colistin, attributed to enhanced outer membrane permeability, induction of oxidative stress, and inhibition of efflux pump activity. This combination therapy significantly improved survival in both *G. mellonella* and murine peritonitis models [231]. In addition to its antimicrobial activity, melatonin functions as a potential host-directed therapy (HDT) due to its potent anti-inflammatory and antioxidant properties. In rodent models of sepsis, melatonin treatment improved survival and ameliorated clinical parameters [232,233]. The HDT mechanisms involve inhibition of the NLRP3 inflammasome, downregulation of pro-inflammatory cytokines, upregulation of anti-inflammatory mediators, and suppression of NF-κB activation by lipopolysaccharide (LPS) [229]. Additionally, melatonin has been proposed to confer nephroprotective effects during colistin therapy [234].

## 5. Repurposing of Drugs for the Treatment of Bioterror Agents

Biothreat pathogens represent a unique subset of infectious threats due to their potential for deliberate dissemination, high mortality rates, and the ability to develop antibiotic resistance, whether naturally or intentionally, to evade standard treatments. The U.S. CDC list of high-priority biological threat agents, known as Category A select agents, includes three bacterial species: *Bacillus anthracis* (Gram-positive), *Yersinia pestis*, and *Francisella tularensis* (both Gram-negative). Category B select agents include *Burkholderia mallei* and *Burkholderia pseudomallei* (both Gram-negative). Until January 2025, Category B also included *Brucella abortus*, *B. melitensis*, and *B. suis*, but these species have since been formally removed from the HHS/USDA Select Agent list and are no longer regulated as select agents by the CDC (https://www.selectagents.gov/regulations/sat-list-changes-2024.htm accessed on 5 May 2025). Current countermeasures rely primarily on conventional antibiotics, which may be rendered ineffective by naturally occurring or engineered multidrug resistance (Table 2). Drug repurposing offers a rapid, cost-effective means to expand the biodefense pharmacopeia by identifying approved drugs with direct or host-directed activity against these pathogens, a strategy suited for emergencies where timely treatment is essential and prompt regulatory and manufacturing responses are required. The sections below summarize repurposed drugs already licensed for other indications that have demonstrated activity against Gram-negative bacteria in Categories A and B.

### 5.1. Plague—Yersinia pestis

Plague is an acute, severe, and contagious disease caused by exposure to *Y. pestis*. In its pneumonic form, untreated infection is invariably fatal. The U.S. Centers for Disease Control and Prevention (CDC) recently updated its treatment guidelines [235]. Recommended antibiotic classes include aminoglycosides (e.g., gentamicin, streptomycin), tetracyclines (e.g., doxycycline), and fluoroquinolones (e.g., ciprofloxacin, levofloxacin), as detailed in Table 2. Antibiotic resistance in *Y. pestis* has been documented, and resistant strains have been isolated from both human cases and infected animals [237]. Moreover, engineered multidrug-resistant strains can be generated with relative ease, underscoring the need for alternative therapeutic options. Consequently, considerable research efforts have been invested worldwide to identify new compounds with activity against resistant *Y. pestis*. For example, an in vitro screen was conducted by our colleagues using a library of >1000 FDA-approved drugs originally developed for oncological, neurological, metabolic, psychiatric, cardiovascular, inflammatory, and infectious indications. Forty-five growth-inhibitory compounds were identified: thirty-seven were known antibiotics active against *Y. pestis*, whereas eight were not previously classified as “antibacterial”. Two anti-cancer agents, bleomycin sulphate and streptozocin, showed potent inhibition of *Y. pestis* growth [243]. Bleomycin is a chemotherapeutic agent commonly used in the treatment of hematological malignancies, such as Hodgkin’s and non-Hodgkin lymphomas, as well as various solid tumors. Streptozotocin is primarily used in the management of pancreatic cancer. Both compounds have demonstrated antimicrobial activity against Gram-negative bacteria [243,244,245]. However, their use as antibacterial agents is not recommended due to unfavorable safety profiles and, in the case of streptozotocin, an exceptionally short plasma half-life [246]. These limitations significantly restrict their potential clinical applicability for infectious disease treatment.

A separate study screened a library of 780 FDA-approved drugs for their ability to prevent macrophage damage following infection with *Y. pestis* [236]. Three repurposed drugs were selected: doxapram (a respiratory stimulant, DXP), amoxapine (an antidepressant, AXPN), and trifluoperazine (an antipsychotic, TFP). All three reduced macrophage injury and inhibited intracellular bacterial replication. In a murine model of pneumonic plague, immediate post-exposure treatment with any of the three agents for three days conferred substantial protection (40–60% survival). This outcome is noteworthy because the compounds lack direct antibacterial activity and do not suppress expression of *Y. pestis* virulence proteins. In a subsequent study, delaying therapy until 24 h post-exposure diminished the efficacy of TFP, however the survival rates following DXP or AXPN were increased as compared to administration at early times post infection. Combining each drug with sub-protective doses of levofloxacin markedly extended the therapeutic window of the antibiotic to 48 h post-exposure. It is important to mention that these drugs demonstrated protective effects also against other bacteria, implicating for a broad-spectrum effect [236,247]. These findings highlight the considerable potential of host-directed therapies (see Section 2).

The value of modulating host responses was also demonstrated in a study conducted by our colleagues that evaluated corticosteroid adjunctive therapy for plague. Dissemination of *Y. pestis* from the portal of entry to the bloodstream triggers a cytokine storm, massive neutrophil infiltration, extensive bacteremia and death. The study tested whether immune-suppressive corticosteroids could mitigate these deleterious reactions. Co-administration of methylprednisolone with sub-optimal therapeutic antibodies significantly enhanced protection in a bubonic plague model, reducing neutrophil accumulation, limiting tissue damage, and promoting more effective bacterial clearance [248]. To prevent progression to sepsis, another investigation assessed prophylactic lovastatin in mice challenged intraperitoneally with *Y. pestis* [249]. Despite lacking direct antibacterial activity, prophylactic lovastatin improved survival, enhanced bacterial clearance from tissues, and attenuated splenic and pulmonary pathology. Table 3 summarizes the compounds described for *Y. pestis* in this section.

### 5.2. Tularemia—Francisella tularensis

Tularemia presents a spectrum of clinical manifestations in humans; the most acute is the pneumonic form, which can follow inhalation of minute infectious doses of *F. tularensis* in dust or aerosol. The CDC currently recommends therapy with aminoglycosides (gentamicin or streptomycin), the fluoroquinolone ciprofloxacin, or the tetracycline antibiotic doxycycline [250], as summarized in Table 2. *F. tularensis* is naturally resistant to many β-lactam antibiotics, and antimicrobial resistance can be artificially introduced via plasmids, underscoring the importance of continued surveillance and preparedness [240]. Relatively few studies have explored alternative treatments for tularemia. Nevertheless, investigations of the pathogen’s virulence mechanisms highlight its highly effective suppression and evasion of host immunity, suggesting that immune pathways themselves constitute attractive therapeutic targets. One strategy, host-directed therapy (HDT; see also Section 2 and Section 5.1), aims to enhance the activity of host immune cells, particularly monocytes and macrophages that serve as an intracellular niche for bacterial replication [251]. A representative example of this approach evaluated imatinib, which at sub-clinical doses increases circulating white-blood-cell counts. Imatinib treatment reduced *F. tularensis* burdens in the spleen and blood of mice infected with the Live Vaccine Strain (LVS) [239,252]. In another study, a high-throughput screen in *C. elegans* was established to assess the anti-tularemia potential of FDA-approved drugs [253]. Diflunisal, a non-steroidal anti-inflammatory drug (NSAID), displayed multiple layers of activity against LVS: direct antibacterial action (MIC = 8 µg/mL), inhibition of intracellular replication in macrophages and epithelial cells at 4 × MIC, and synergism with ciprofloxacin in macrophages, together with an encouraging in vitro safety profile. Mefloquine, an antimalarial drug, also emerged from the screen but exhibited a higher MIC (16 µg/mL), and its evaluation did not progress beyond MIC determination. Paroxetine, a selective serotonin-reuptake inhibitor (SSRI) used to treat depression, improved *C. elegans* survival after *F. tularensis* infection, indicating antibacterial activity (see Section 3.14 for additional details on SSRIs and paroxetine) [253].

The NSAID celecoxib, a selective cyclooxygenase-2 (COX-2) inhibitor, exhibited antibacterial activity against several *F. tularensis* subspecies (SchuS4, LVS, and *F. novicida*; MIC = 16–32 µg/mL). A specific chemical derivative of celecoxib inhibited intracellular growth in macrophages at an MIC of 4 µg/mL and has been proposed for further development. The antibacterial effect appeared to be independent of the drug’s anti-inflammatory effect, as rofecoxib, a more potent COX-2 inhibitor, showed no activity against *F. tularensis* [254]. Table 4 provides a summary of the agents presented for *F. tularensis*.

### 5.3. Melioidosis—Burkholderia pseudomallei

Melioidosis is caused by the environmental Gram-negative bacterium *Burkholderia pseudomallei*, which inhabits surface waters and soils. The disease is endemic to tropical regions of South-East Asia (e.g., Thailand, India), northern Australia, and parts of Africa (e.g., Nigeria); in recent years, locally acquired cases have also been confirmed along the Gulf Coast of Mississippi, USA, indicating the bacterium’s establishment in the environment [255]. Recommended therapy is prolonged: ≈2 weeks of parenteral antibiotics followed by 3 months of oral treatment [256]. According to the CDC, first-line agents include trimethoprim–sulfamethoxazole, ceftazidime, and amoxicillin/clavulanic acid, as summarized in Table 2. Recent reports increasingly describe antibiotic-resistant *B. pseudomallei* strains [257]. *B. pseudomallei* is intrinsically resistant to multiple antibiotic classes, largely attributed to the activity of multiple drug-efflux pumps [241,257]. Given the pathogen’s environmental reservoir and potential for aerosolization, drugs with both intracellular penetration and activity under biofilm conditions warrant prioritization. A study testing this hypothesis examined whether efflux-pump inhibitors could restore susceptibility in resistant strains [258]. Phenothiazine derivatives, prochlorperazine, chlorpromazine, and promazine, used clinically as antihistamines or antipsychotics, produced marked synergy with a wide range of aminoglycosides and macrolides. Combination treatment dramatically lowered MIC values for streptomycin, erythromycin, spectinomycin, levofloxacin, azithromycin, and amoxicillin/clavulanic acid [258]. In a separate repurposing effort, 400 FDA-approved drugs (Pathogen Box) were screened for bacteriostatic or bactericidal activity against *B. pseudomallei* [259]. Auranofin has shown in vitro antibacterial activity against *B. pseudomallei*, albeit at relatively high concentrations (MIC = 150 µg/mL) [259] (see Section 3.7 for broader discussion of auranofin’s antibacterial and adjuvant properties against Gram-negative pathogens). To explore adjunctive modulation of the dysregulated host immune response (“cytokine storm”) characteristic of severe melioidosis, the selective COX2 inhibitor tolfenamic acid (TA), clinically used for migraine and acute pain, was evaluated [260]. Co-administration of TA with a sub-therapeutic dose of ceftazidime significantly improved survival in a murine inhalational model, relative to antibiotic monotherapy, and produced a marked reduction in pulmonary bacterial load [260]. Another study assessed the potential of IFN-γ immunotherapy to enhance conventional treatment of *B. pseudomallei* infection. In vitro and in vivo models showed that even low doses of IFN-γ combined with ceftazidime produced strong synergistic inhibition, reduced bacterial burden, and limited dissemination. These findings suggest that IFN-γ may potentiate antimicrobial therapy in acute melioidosis [261]. Table 5 summarizes the therapeutic candidates discussed for *B. pseudomallei*.

## 6. Discussion and Outlook

Drug repurposing represents a pragmatic and cost-effective adjunct to the waning antibiotic pipeline. Evidence reviewed here demonstrates that diverse classes of non-antibiotic drugs possess antibacterial or anti-virulenceactivities against Gram-negative pathogens.

Equally significant is the host-directed potential of many repurposed drugs. By modulating immune pathways, controlling inflammation, and accelerating bacterial clearance, these agents provide dual modes of action. Their application may be particularly relevant in scenarios involving pathogens exploited as biological warfare agents, where rapid deployment of existing safe drugs could mitigate catastrophic outcomes.

An additional important aspect relates to antimicrobial stewardship. Beyond their direct antibacterial or host-directed activities, several repurposed agents act as antibiotic adjuvants, thereby restoring the efficacy of conventional antibiotics and reducing the risk of resistance development during therapy. By lowering the selective pressure associated with high-dose monotherapy and limiting the emergence of resistant subpopulations, repurposed drugs can extend the clinical utility of existing agents. Moreover, immunomodulatory repurposed drugs, such as statins, may enhance bacterial clearance while attenuating harmful inflammation, offering further benefits within stewardship frameworks. Thus, incorporating repurposed drugs into therapeutic strategies not only expands treatment options but also supports long-term preservation of last-line antibiotics. The potential mass use or emergency deployment of repurposed drugs also raises regulatory, ethical, and access-related considerations. Ensuring equitable availability and affordability, particularly in low-resource settings, will be essential for their effective integration into global health responses.

While antibacterial treatment with repurposed drugs is promising, all reported activities in this review are currently supported only by in vitro or preclinical data, and clinical validation remains an essential next step. Where available, evidence of clinical relevance has been cited within the text. In this regard, although the translational value of in vitro and preclinical findings is often uncertain, many of the compounds discussed, such as dietary supplements, are known to have favorable safety profiles, supporting their potential inclusion in antibacterial regimens even if not sufficient alone. Additionally, even when clinically achievable concentrations are limited, combination therapies involving several repurposed agents may still enhance antibacterial efficacy. In summary, advancing these candidates toward clinical use will require clinical trials, formulation optimization, definition of synergistic dosing strategies with existing antibiotics, and comprehensive pharmacokinetic and pharmacodynamic evaluation within infectious disease settings.

In this regard, it is noteworthy that, to the best of our knowledge, clinical trials involving repurposed antibacterial agents remain limited. This scarcity may be attributed, at least in part, to the lack of commercial incentives associated with off-patent compounds.

## 7. Conclusions

Repurposing of approved non-antibiotic drugs offers a timely opportunity to address the AMR crisis in Gram-negative bacterial infections. By highlighting both direct antibacterial properties and host-directed effects, this review underscores the therapeutic potential of agents repurposed from multiple pharmacological domains. Integration of these approaches, whether through synergistic antibiotic combinations, host-directed therapy, or emergency preparedness against resistant biothreat pathogens, can significantly broaden our clinical armamentarium against MDR Gram-negative infections. Continued translational research and well-designed clinical trials will determine how these old drugs can define new frontiers in the fight against resistant bacterial pathogens.

## Figures and Tables

**Table 2:** Category A/B Gram-negative bacterial agents, current standard-of-care therapies, and AMR concerns.

Pathogen	CDC Category	Current Standard Therapy (CDC)	Known/Potential Resistance
*Yersinia pestis*	A	Gentamicin or fluoroquinolones (e.g., ciprofloxacin, levofloxacin); sometimes streptomycin or doxycycline (IV or PO, 10–14 days) [235,236]	Plasmid-mediated resistance can be acquired naturally and artificially; gene point mutations [237]
*Francisella* *tularensis*	A	Gentamicin (5 mg/kg IM/IV, 10–14 days), ciprofloxacin (400 mg IV or 500 mg PO twice daily, 10–14 days), doxycycline (100 mg IV/PO twice daily, 14–21 days) https://www.cdc.gov/tularemia/hcp/clinical-care/index.html accessed on 5 May 2025) [238,239]	Naturally resistant to many β-lactams; antimicrobial resistance can be artificially introduced with plasmids [240]
*Burkholderia pseudomallei*	B	Intensive phase: IV ceftazidime every 6–8 h or meropenem every 8 h; eradication phase: TMP-SMX (with folic acid), or co-amoxiclav, or doxycycline for 3–6 months CDC—Melioidosis Clinical Overview: https://www.cdc.gov/melioidosis/hcp/clinical-overview/index.html accessed on 5 May 2025).	Intrinsic resistance to many antibiotics via efflux pumps; biofilm-associated tolerance [241,242]
*Burkholderia mallei*	B	Similarly to *B. pseudomallei*	Similarly to *B. pseudomallei*; fewer clinical data

**Table 3:** *Yersinia pestis*.

Drug	Original Indication	Mechanism	Evidence	Advantages	Limitations
Bleomycin	Oncology (Hodgkin’s, NHL, solid tumors)	Direct	In vitro growth inhibition [243]	Broad Gram-negative activity	High toxicity; IV only
Streptozotocin	Oncology (pancreatic cancer)	Direct	In vitro activity [243]	Potent vs. resistant strains	Very short t½; toxicity
Doxapram	Respiratory stimulant	HDT	Murine pneumonic plague; ±levofloxacin synergy [236]	Extends antibiotic window; broad activity	Limited infection clinical data
Amoxapine	Antidepressant (TCA)	HDT	Murine pneumonic plague; ±levofloxacin [236]	Inhibits intracellular replication	CNS side effects
Trifluoperazine	Antipsychotic	HDT	Early post-exposure protection in mice [236]	Reduces macrophage injury	Efficacy reduced with delayed start
Lovastatin	Hyperlipidemia	HDT	Murine prophylaxis improved survival [249]	Anti-inflammatory; less pathology	Tested prophylactically
Methylprednisolone	Corticosteroid	HDT (adjunct)	Adjunct to antibodies improved outcomes [248]	Limits tissue damage	Immunosuppression risk

**Table 4:** *Francisella tularensis*.

Drug	Original Indication	Mechanism	Evidence	Advantages	Limitations
Imatinib	Oncology (CML, GIST)	HDT	Murine LVS: decreased bacterial load [239,252]	Boosts myelopoiesis/WBCs	Oncology-grade toxicities
Diflunisal	NSAID	Direct + HDT	MIC; macrophage activity; synergy with ciprofloxacin [253]	Multi-layered activity	NSAID toxicity (chronic)
Mefloquine	Antimalarial	Direct	In vitro MIC ≈ 16 µg/mL [253]	Intracellular penetration	Neuropsychiatric AEs
Paroxetine	SSRI	Direct (screen)	Increased *C. elegans* survival vs. LVS [253]	Known safety profile	Limited antibacterial potency data
Celecoxib derivatives	NSAID (COX-2)	Direct (independent of COX-2)	In vitro; macrophage growth inhibition [254]	Anti-inflammatory + antibacterial	Parent drug weaker

**Table 5:** *Burkholderia pseudomallei*.

Drug	Original Indication	Mechanism	Evidence	Advantages	Limitations
Phenothiazines (prochlorperazine, chlorpromazine, promazine)	Antipsychotic/antihistamine	Direct (efflux-pump inhibition)	Synergy with aminoglycosides & macrolides; decreased MICs [258]	Restores activity of multiple classes	CNS/cardiac AEs; repurposing dose/PK gaps
Auranofin	Rheumatoid arthritis	Direct	In vitro MIC ≈ 150 µg/mL [259]	Broad Gram-neg. potential	High MIC; formulation issues
Tolfenamic acid	NSAID (migraine/pain)	HDT	Murine inhalational model: synergy with ceftazidime; decreased lung burden [260]	Adjunctive benefit; immune modulation	Not widely available everywhere
Interferon-γ	Immunomodulatory therapy (adjunct use)	HDT	Preclinical: in vitro + murine models [261]	Synergy, intracellular clearance, reduced relapse	Safety and dosing unknown
(IFN-γ)		(production of reactive oxygen species)			

## Data Availability

No new data were created or analyzed in this study. Data sharing is not applicable to this article.
